# “Polyradiculoneuritis” as an Atypical Clinical Presentation of Creutzfeldt–Jakob Disease: A Case Report and Review of Literature

**DOI:** 10.3390/life16040684

**Published:** 2026-04-17

**Authors:** Elisa Colaizzo, Anna Ladogana, Dorina Tiple, Luana Vaianella, Giuseppe Bufano, Fabio Moda, Daniela Merlo, Eloise Longo, Alessia Perna

**Affiliations:** 1 Department of Neuroscience, Istituto Superiore di Sanità, Viale Regina Elena 299, 00161 Rome, Italy; elisa.colaizzo@iss.it (E.C.); anna.ladogana@iss.it (A.L.); dorina.tiple@iss.it (D.T.); luana.vaianella@iss.it (L.V.); daniela.merlo@iss.it (D.M.); eloise.longo@iss.it (E.L.); 2 Unit of Laboratory Medicine, Laboratory of Clinical Pathology, Fondazione IRCCS Istituto Neurologico Carlo Besta, Via Celoria 11, 20133 Milan, Italy; giuseppe.bufano@istituto-besta.it (G.B.); fabio.moda@istituto-besta.it (F.M.); 3 Department of Medical Biotechnology and Translational Medicine, University of Milan, Via Fratelli Cervi 93, 20054 Milan, Italy; 4 USMAF-SASN Fiumicino, Italian Health Ministry, Via Mario Stoppani, 20, 00054 Fiumicino, Italy

**Keywords:** polyradiculoneuritis, CJD atypical presentation, rare CJD phenotype, CJD-related neuropathy, numbness, paresthesia

## Abstract

(1) Background: Creutzfeldt–Jakob disease (CJD) is a progressive neurodegenerative disorder, characterized by cognitive decline, and motor and psychiatric symptoms; it primarily affects the central nervous system; however, peripheral nervous system involvement has rarely been described, particularly as an atypical presentation. (2) Methods: A 78-year-old Caucasian man, a retired farmer with no family history of neurological disease, presented with diarrhea followed by progressive lower limb weakness, which eventually evolved into encephalopathy and generalized areflexia. An initial diagnosis of inflammatory neuropathy was considered; the diagnostic assessment included blood and cerebrospinal fluid testing, a CT whole body scan, brain MRI, neuropsychological testing, electroencephalography, a nerve conduction study and electromyography. (3) Results: Neurophysiological studies demonstrated an acute asymmetrical sensorimotor, predominantly axonal polyneuropathy, initially suggestive of an axonal form of inflammatory polyradiculoneuritis. This pattern was confirmed on follow-up neurophysiological assessment performed three weeks later. Unexpectedly, the diagnostic course ultimately led to a diagnosis of sporadic Creutzfeldt–Jakob disease, confirmed by post-mortem neuropathological examination. Based on these findings, we conducted a literature review to summarize the current evidence on CJD-related neuropathy. (4) Conclusions: Our case emphasizes the importance of maintaining clinical suspicion for CJD even in patients presenting with progressive lower limb weakness and suggests that peripheral neuropathy may be concomitant or even precede the CNS manifestations. Careful consideration is required to avoid misdiagnosis of inflammatory neuropathy in the context of neurodegenerative diseases such as CJD.

## 1. Introduction

Creutzfeldt–Jakob disease (CJD) is a rare and fatal neurodegenerative disorder belonging to the group of prion diseases, caused by the accumulation of a misfolded prion protein, the scrapie prion protein (PrP^Sc^), within the central nervous system (CNS) [1].

Initially, PrP^Sc^ deposition was thought to be restricted in the CNS. However, subsequent studies have demonstrated the extraneural deposition of PrP^Sc^ in the spleen, olfactory-nerve tissue, muscle samples and other systemic organs [2,3]. Following the introduction of the Real-Time Quaking-Induced Conversion (RT-QuIC) method for the diagnosis of human prion diseases, the PrP seeding activity has been definitely demonstrated in peripheral tissues, including in peripheral nerves [4].

Its clinical presentation can vary widely, ranging from rapidly progressing dementia—often associated with psychiatric manifestations and behavioral changes—to gait ataxia, peripheral neuropathy, and autonomic nervous system dysfunction, thereby posing significant challenges in differential diagnosis [1].

Here, we report a case of sporadic CJD presenting with early peripheral neuropathy in a 78-year-old patient. In this regard, the use of a follow-up nerve conduction study (NCS) and electromyography (EMG) for CJD diagnosis has not been widely reported [5].

Conversely, in the early stages of the disease, the interpretation of NCS/EMG findings can be difficult; follow-up NCS/EMG studies may be important for differential diagnosis and facilitating the timely initiation of appropriate therapy [6].

## 2. Case Report

### 2.1. Clinical Summary

A 78-year-old Caucasian male patient, a retired farmer, with diabetes mellitus and arterial hypertension presented with a 15-day history of diarrhea, followed by rapidly progressive gait instability. His family history was negative for neurological diseases. Neurological examination revealed waddling gait, paresthesia and hypopallesthesia in the lower limbs, along with a positive Mingazzini’s maneuver.

Comprehensive peripheral blood testing, including an extensive autoimmune assessment panel, was within the normal ranges, except for a markedly elevated prostate-specific antigen (PSA). Cerebrospinal fluid (CSF) analysis documented mild pleocytosis (white cell count of 15.2 × 10^9^/L), but CSF culture and cytological results were negative. Also serological tests for neurosyphilis (VDRL), cytomegalovirus (CMV), hepatitis B virus (HBV), hepatitis C virus (HCV), human immunodeficiency virus (HIV), Epstein–Barr virus (EBV) and Toxoplasma Gondii were negative. Tumor markers, antiganglioside antibodies and onconeural antigens were also negative. The diagnostic workup included CT whole body computed tomography, 1.5-T brain magnetic resonance imaging (MRI), neuropsychological assessment, electroencephalography (EEG), a nerve conduction study (NCS) and electromyography (EMG). Based on the clinical examination, and to exclude other diseases, the patient underwent a first NCV/EMG performed one month after onset. Given the NCS/EMG findings of acute asymmetrical sensorimotor axonal peripheral neuropathy, an inflammatory peripheral neuropathy likely “polyradiculoneuritis” was considered.

Consequently, the patient was treated with intravenous prednisolone (1 g/day) and immunoglobulins (IVIG) at a dose of 0.4 g/kg/day for five consecutive days, without any clinical improvement. Repeat NCV/EMG performed three weeks later showed further axonal deterioration. In the meantime, the patient developed spatiotemporal disorientation and visual hallucinations, associated with dysarthria, bilateral dysmetria and ataxia affecting both upper and lower limbs. Distal sensory deficits became more pronounced, particularly on the right side, and progressive muscle atrophy was noted. Neuropsychological tests confirmed mild global cognitive impairment, with deficits in long-term verbal memory, mild short-term memory impairment, and apathy. The score on the Mini-Mental State Examination (MMSE) was 22/30 [7].

An electroencephalogram (EEG) demonstrated theta activity and periodic synchronous discharges (PSD). Based on these typical findings, prion disease was suspected, and the CSF tested positive for the 14-3-3 protein, likewise testing positive in RT-QuIC analysis [8].

Meanwhile, the patient experienced myoclonus and clinical decline, progressing rapidly into akinetic mutism. He died three months after neurologic onset.

### 2.2. Methods

#### 2.2.1. Biochemical Analysis of Brain Areas

Frozen brain tissue from the cerebellum and cingulate gyrus was homogenized in cold lysis buffer (100 mM NaCl, 10 mM EDTA, 0.5% Nonidet P-40, 0.5% sodium deoxycholate in 10 mM Tris-HCl, pH 7.4). The homogenates were digested with proteinase K (50 µg/mL, 1 h at 37 °C), and the enzymatic activity was terminated by boiling the samples in loading buffer (Bolt LDS Sample Buffer and DTT, Thermo Scientific US, Waltham, MA, USA; California Pall Corporation Italy, Milano; BMG Labtech Germany, Ortenberg) at 100 °C for 10 min. The samples were then subjected to Western blot (Wb) analyses, and the PVDF membrane was probed with the 3F4 antibody. After incubation with a secondary antibody (Fab fragment anti-mouse IgG conjugated with HRP, GE), the membrane was developed using the ECL Prime detection system (Amersham) and chemiluminescence was visualized using a G:BOX Chemi Syngene system.

#### 2.2.2. RT-QuIC Assay

Recombinant truncated Syrian hamster PrP (recSHaPrP_90–231_) was thawed and filtered through a 100 kDa Nanosep centrifugal device (Pall Corporation). The RT-QuIC reaction mixture consisted of 10 mM PBS (pH 7.4), 150 mM NaCl, 0.13 mg/ml recSHaPrP_90–231_, 1 mM EDTA, 0.002% SDS and 10 μM thioflavin T (ThT). A 93 μL volume of the reaction mix was dispensed into each well of a black 96-well optical flat bottom plate (Thermo Scientific) and supplemented with 7 μL of CSF. Each sample was analyzed in quadruplicate. The plate was sealed with a sealing film (Thermo Scientific) and subjected to intermittent cycles of shaking (1 min at 600 rpm, double orbital) and incubation (1 min) at 55 °C using the fluorescence microplate reader OMEGA (BMG Labtech). ThT fluorescence was measured every 30 min (wave-lengths: excitation 450 ± 10 nm; emission 480 ± 10 nm). A sample was considered positive when at least 2 of 4 replicates exceeded 10,000 arbitrary units (AU) within the 60 h time threshold.

#### 2.2.3. Nerve Conduction Study and Electromyography

Motor nerve conduction studies were performed on the fibular and posterior tibial and median, left ulnar nerves, with recording of compound muscle action potential (CMAP). Sensory nerve conduction studies were initially conducted by assessing sensory action potentials (SAPs) in the left median, ulnar and radial nerves as well as the sural nerve bilaterally. For each CMAP, the amplitude, duration, and area of the negative peak were measured. F-waves were performed for each nerve to determine the minimal F-wave latency or to document F-wave absence. Electromyography was carried out using standard concentric needle electrodes on lower limb muscles and distal upper limb muscles. For each CMAP, the amplitude, duration, and area of the negative peak were measured. F-wave studies were conducted for each nerve to determine the minimal F-wave latency or to document F-wave absence.

### 2.3. Results

#### 2.3.1. Imaging

**Figure 1:**
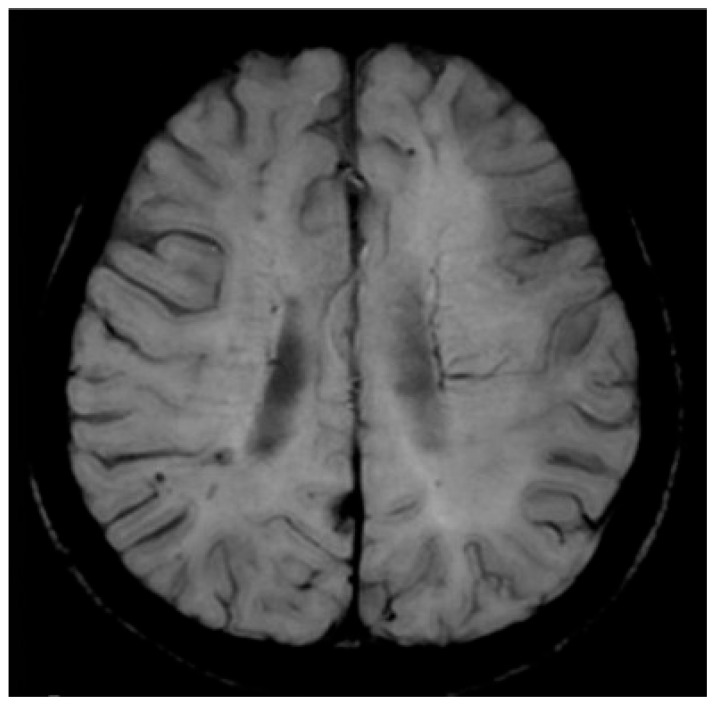
Brain CT imaging showed no acute abnormalities.

A computed tomography (CT) scan of the brain was normal (Figure 1).

A total body CT was performed under the suspicion of a paraneoplastic pathology, but showed nothing relevant. During hospitalization, the patient underwent two brain magnetic resonance imaging (MRI) scans, which showed a progressive DWI hyperintensity signal on the bilateral frontal mesial cortex, and on the putamen and caudate nucleus, mostly on the right.

#### 2.3.2. Western Blot Analysis

**Figure 2:**
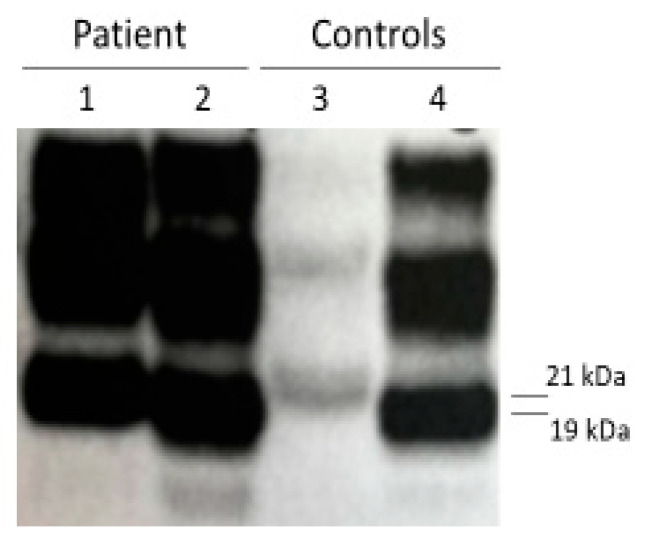
Western blot analysis of brain homogenates showing distinct PrP^res^ types. The cingulate gyrus displays a type 1 PrP^res^ profile, whereas the cerebellum exhibits a type 2 PrP^res^ pattern. Molecular typing was performed after PK digestion and immunoblotting with anti-PrP antibodies. Legend: 1. Gyrus cinguli; 2. Cerebellum; 3. PrP^res^ type 1; 4. PrP^res^ type 2.

Immunoblot analysis revealed distinct PrP^res^ profiles in the two brain regions examined [9] (Figure 2).

**Figure 3:**
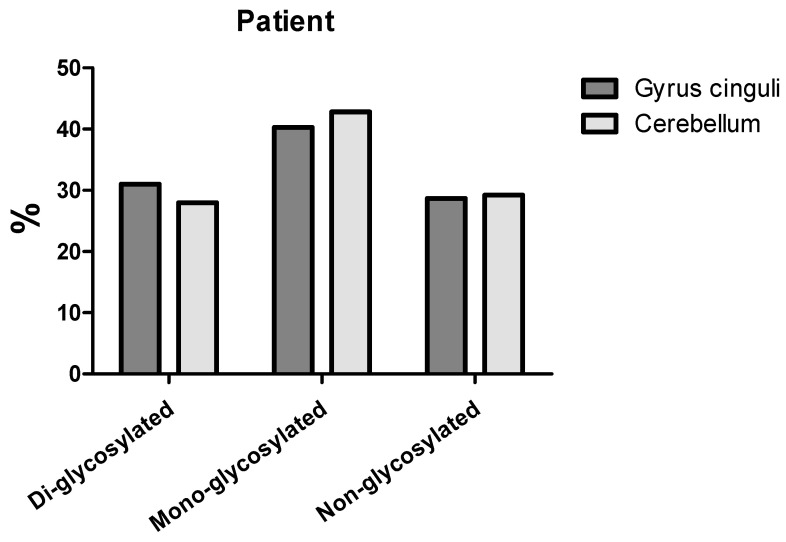
Densitometric analysis of PrP^Sc^ species analyzed in the brain. Histograms showing the signal intensity of each PrP^Sc^ band (di-, mono- and non-glycosylated) after the Western blot analysis of the two brain areas. The predominance of the mono-glycosylated band confirms the CJD-associated biochemical profile.

In the cingulate gyrus, PrP^res^ type 1 was detected, whereas the cerebellum displayed a PrP^res^ type 2 pattern Figure 3.

The coexistence of different PrP^res^ types within the same patient has been previously reported and is thought to reflect either regional heterogeneity of prion replication or the simultaneous presence of mixed strain populations (10.1038/s41598-020-58446-0; 10.1212/wnl.53.9.2173).

#### 2.3.3. RT-QuIC Analysis

**Figure 4:**
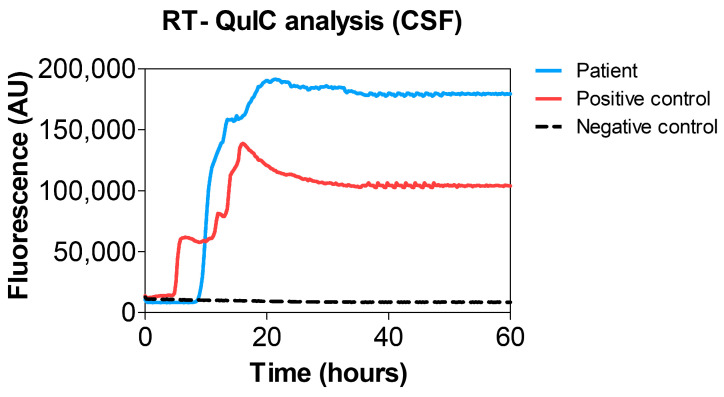
RT-QuIC analysis of the CSF. RT-QuIC fluorescence kinetics of CSF samples using recSHaPrP_90–231_ as substrate. The curves show (i) the patient CSF (light blue line), (ii) the CSF used as a positive control (red line) and (iii) the CSF used as a negative control (black dotted line).

In addition, RT-QuIC analysis of cerebrospinal fluid yielded a positive result, confirming the presence of PrP seed-competent aggregates (Figure 4).

#### 2.3.4. Nerve Conduction Study and Electromyography

The initial NCV/EMG demonstrated abnormalities in both sensory and motor segments of the lower extremities, including F-waves, consistent with a patchy axonal neuropathy with mild conduction slowing. Specifically, the left sural, left ulnar sensory response and left fibular motor responses were absent. The left superficial radial nerve was reduced, whereas the right sural and right fibular motor responses showed mild slowing despite preserved amplitudes. F-wave responses were normal in the left upper limb and unrecordable in the lower limb, although the right fibular motor response amplitude was within normal limits. Follow-up evaluation (repeated three weeks later) confirmed disease progression, with further axonal involvement, particularly affecting both upper and lower limbs, while the conduction velocities remain mildly delayed in the lower limbs. Needle electromyography of tibialis anterior and gastrocnemius showed reduced motor unit recruitment and mild neurogenic changes (Table 1).

## 3. Discussion

The initial neurophysiological findings suggested a multifocal, mixed sensorimotor neuropathy that is predominantly axonal in nature and supported by a patchy distribution in severity. This pattern is suggestive of an inflammatory origin of symptoms.

Although the patient had diabetes mellitus, which is a known risk factor for peripheral neuropathy, the electrophysiological features observed were not consistent with typical diabetic neuropathy, which usually presents as a symmetrical length-dependent process. Instead the observed pattern was patchy, asymmetric, and non-length-dependent, supporting an alternative etiology [10].

In addition, the acute onset of symptoms and marked asymmetry further argue against a diabetic aetiology and instead support the initial diagnosis of acquired inflammatory neuropathy. However, the patient’s neuropathy did not respond to the immunosuppressive therapy. Although the disease is classically confined to the central nervous system, several case reports and small series have documented peripheral neuropathy as an early or even presenting feature. Both axonal and demyelinating patterns have been reported, often mimicking inflammatory neuropathies such as chronic inflammatory demyelinating polyradiculoneuropathy (CIDP) or acute inflammatory polyradiculoneuritis.

We conducted the literature review as follows: the PubMed database was searched using combinations of the keywords “Creutzfeldt–Jakob disease” or “prion” and “peripheral neuropathy” or “demyelinating neuropathy”. At the time of writing (1 January 2026), only English-language publications were considered (publication years: 1970–2026). A total of 27 articles were accessible for full-text review and were extensively analyzed to collect relevant clinical, biochemical, genetic, and/or neuropathological data.

The occurrence of peripheral neuropathy at CJD onset was previously reported in 4% of 158 CJD patients in a retrospective study in England and Wales starting from 1970 [11].

Intriguingly, in 1996 Antoine et al. described a patient with Glu200Lys mutation, presenting with demyelinating peripheral neuropathy, cerebellar ataxia, subcortical dementia and rigidity [12].

To date, this unusual CJD presentation has been observed not only in genetic forms, but also in sporadic cases. Interestingly, Baiardi et al. examined over 300 medical records of sCJD and found a higher prevalence of peripheral nervous system (PNS) involvement in sCJDMV2K and VV2 subtypes, suggesting a prion strain-specific effect [13]. A significantly higher prevalence of PNS involvement in genetic E200K CJD patients (14.6% vs. 5.6% respectively, *p* = 0.0005) was subsequently reported by Appel et al., who by screening the CJD Israeli National Database found that the most common sensory symptoms were numbness and neuropathic pain [14]. Similarly, Neufeld diagnosed two Jewish Libyan patients with mutation of codon 200 in the prion protein, who manifested signs of peripheral nerve involvement [15].

Zeidler et al. described 8/21 nvCJD cases presenting pain, paresthesia and/or dysesthesia in the hands, feet, mouth, and even the lumbar region; in four of these cases, severe and persisting sensory symptoms preceded the psychiatric symptoms [16]. Unfortunately, since these sensory symptoms are indistinguishable from other etiologies (e.g., Guillain-Barré Syndrome and CIDP, chronic inflammatory demyelinating polyneuropathy), a late and incorrect diagnosis of CJD neuropathy at onset may lead to ineffective, expensive and unnecessary immunomodulatory treatment and/or invasive therapy (e.g., plasmapheresis), as previously documented [17,18].

Although neuropathy at CJD onset is frequently underrecognized and in any case unpredictable, it is crucial to not completely exclude it from the differential diagnosis to avoid significant diagnostic delays. Even though CJD is a rare disease with a generally progressive clinical course, it is essential to gather and enhance the understanding of all early-stage symptoms, even if non-cognitive and/or limited to a single domain at disease onset. Despite the fact that nerve conduction results are nonspecific, and it is often very difficult to diagnose sCJD in the antemortem stage, RT-QuIC, EEG and MRI findings can give clinicians the opportunity to promptly recognize and diagnose even the more atypical and unusual CJD presentations, before neuropathological confirmation.

Overall, previous studies have already highlighted the possible involvement of the PNS in the pathogenesis of transmissible spongiform encephalopathies (TSEs) [19], furthermore underscoring the need to understand whether abnormal neurophysiological findings may represent a marker of disease progression during therapeutic trials [20].

More recently, in 2002, Niewadomska et al. [21] performed electrophysiologic examinations on 15 consecutive sCJD patients. They found axonal-demyelinating neuropathy in 4 cases, axonal disease in 9 patients, and 1 patient with motor neuron disease. They concluded that 88% of these patients showed damage to the PNS [22]. In the same year, Follet et al. demonstrated that Schwann cells also express the prion protein PrP^C^ and can support the replication of the infectious PrP^Sc^, proving that they are crucial for the spread of prion diseases from the periphery to the CNS. They found PrP^C^ on Schwann cell membranes in mice and showed that an infected glial cell line (MSC-80) could produce PrP^Sc^ and transmit the disease to mice, thus filling a gap in understanding how prions travel from peripheral infection sites to the brain.

Lastly, Cardone et al. investigated the process of prion spread from the site of infection to the CNS, by in vivo exposure/injection of sciatic nerves with 263 K scrapie prions in golden Syrian hamsters (GSHs). They demonstrated that prion entry likely occurs via peripheral routes and projects to the CNS via peripheral nerves. Furthermore, the rate of prion spread along peripheral nerves may be faster than previously reported. This experimental approach could allow verification of the timing of neuroinvasion, a relevant parameter for determining possible therapeutic interventions [23]. Furthermore, Chen and colleagues showed that prions can reach the nervous system by gaining access along peripheral nerves via macropinocytosis with varying efficiency and timing of neuroinvasion and intraneural propagation [24]. Similarly, Delorme et al. [25] described a heterogeneous pattern of demyelination with motor blocks—mimicking CIDP—in two unrelated CJD patients with the E200K mutation. Interestingly, they observed that CJD-related neuropathy could even appear up to two years before CNS involvement, making it easily misdiagnosed as CIDP or a late-onset form of CMT disease. Moreover, they argued that PrP^Sc^ can be deposited in the PNS and propagate to the brain in sporadic forms of CJD.

CJD was also suspected in a 60-year-old Caucasian male who presented with painful sensorimotor neuropathy, ophthalmoparesis and ataxia, misdiagnosed as Miller Fisher syndrome, and unsuccessfully treated with plasmapheresis. Follow-up MRI and EEG revealed the typical CJD findings [26]. In another case of chronic inflammatory demyelinating neuropathy, reported by Weckhuysen et al., [27] an initial favorable response to IV immunoglobulins led to a diagnostic pitfall of CIDP. However, after a few months, the patient developed cognitive impairment associated with cerebellar ataxia, and neuropathological analysis revealed spongiform changes, proliferation and hyperplasia of the astrocyte glia in the cerebellar cortex. Genetic analysis revealed a p.Glu200Lys mutation in the PRNP gene.

The principal limitation of our study is the lack of peripheral nerve or other organ samples for neuropathological examination, to detect abnormal PrP deposits and collect histological features [28].

On the other hand, our case highlights that peripheral nervous system involvement at CJD onset is very uncommon and may represent a diagnostic challenge.

Importantly, these peripheral manifestations can closely resemble inflammatory neuropathies, leading to potential misdiagnosis and inappropriate initiation of immunotherapy. Several reports emphasize that CJD may present with a CIDP-like or polyradiculoneuritis-like phenotype, particularly in the early stages, before the emergence of the characteristic rapidly progressive encephalopathy [29].

From a pathophysiological perspective, the mechanisms underlying peripheral involvement remain incompletely understood. Abnormal prion protein deposition has been demonstrated in dorsal root ganglia and, to a lesser extent, peripheral nerves, although this does not consistently correlate with clinical neuropathy. This suggests that peripheral dysfunction may result from a combination of direct prion-related toxicity and secondary neurodegenerative processes rather than a true inflammatory mechanism [30]. 

Overall, the available literature supports the concept that peripheral neuropathy in CJD is rare but well-documented, and heterogeneous in presentation, and may represent a source of diagnostic confusion, particularly in the early stages of diseases. Awareness of this association is essential, particularly when neurophysiological findings suggest an inflammatory neuropathy in the context of a rapidly evolving neurological syndrome [31].

## 4. Conclusions

This case highlights an atypical presentation of sporadic Creutzfeldt–Jakob disease, in which early neurophysiological findings suggested a peripheral neuropathy, leading initially to consideration of an inflammatory neuropathy. Nerve conduction studies and electromyography consistently demonstrated an asymmetrical, predominantly axonal sensorimotor polyneuropathy with a non-length-dependent distribution, a pattern typically suggestive of an acquired inflammatory process such as axonal polyradiculoneuritis.

However, the subsequent clinical evolution was not compatible with an immune-mediated peripheral neuropathy. Instead, the rapidly progressive neurological decline, together with supportive ancillary investigations, ultimately led to a central neurodegenerative process, confirmed post-mortem.

Peripheral nervous system involvement is sporadic although uncommon, and remains incompletely characterized, despite many cases being reported in the literature. The disease is classically defined by rapidly progressive dementia, myoclonus, cerebellar dysfunction, and pyramidal/extrapyramidal signs, reflecting widespread central nervous system involvement. In contrast, neurophysiological evidence of peripheral neuropathy is rarely reported and, when present, is typically overshadowed by the ruling central features.

In this context, the recognition of a predominantly axonal, asymmetric neuropathy raises important considerations. Proposed mechanisms include secondary axonal degeneration due to central neuronal loss, anterior horn cell involvement, or less commonly, direct prion-related pathology affecting peripheral nerves.

From a clinical perspective, this case highlights a significant diagnostic pitfall. Early prominent peripheral neurophysiological abnormalities may lead to misclassification as an inflammatory neuropathy, particularly when supported by features such as asymmetry, axonal involvement, and apparent progression on serial studies. However, the absence of response to immunotherapy and the rapid progression of neurological decline should prompt reconsideration of the diagnosis.

Ultimately, this case emphasizes that atypical peripheral neurophysiological findings do not exclude a diagnosis of sporadic CJD.

## Figures and Tables

**Table 1:** Nerve conduction studies; abnormal values in bold.

Motor Nerves	Latency (ms)		Amplitude (mV)		Conduction Velocity (m/s)		F-Waves (ms)	
Left Median	First	Second	First	Second	First	Second	First	Second
Wrist—APB	**3.7**	3.1	8.5	**6.0**			26.8	30.6
Elbow—wrist	9.1	8.1	7.4	**5.3**	48.1	48		
Left Ulnar								
Wrist—ADM	2.8	2.6	9.4	**5.6**			28.0	30.4
Bel elb-wrist	8.1	8.0	8.1	**5.1**	**47.2**	**47.2**		
Ab elb—bel elb	9.9	10.1	7.7	**4.6**	**44.4**	**45.2**		
Right Tibial								
Ankle—Ab Hal	4.1	3.6	**1.5**	**0.6**			**Absent**	**Absent**
Popl-Ankle	17.4	14.5	**1.4**	**0.7**	**33.1**	**36.7**		
Right Fibular								
Ankle—EDB	4.6	4.6	3.1	**0.4**			**Absent**	**Absent**
Fib head—Ankle	13	12.5	2.4	**0.4**	**35.0**	**38.6**		
Left Fibular								
Ankle—EDB	5.2	**NR**	**0.9**	**NR**			**Absent**	**Absent**
Fib head—ankle	14.9		**0.7**		**32.1**			
Sensory Nerves:								
Left radial								
Forearm—thumb	2.5	2.0	**6.0**	**4.7**	51.0	**45.0**		
Right Sural								
Calf—ankle	3.4	**2.5**	**2.0**	**2.2**	**32.4**	**33**		
Left Sural								
Calf—ankle	**NR**	**NR**						
Left Ulnar								
Wrist—Dig V	**NR**	**NR**						

Abbreviations: Ab elb—above elbow; bel elb—below elbow; ADM—abductor digiti minimi; APB—abductor pollicis brevis; dig—digit; EDB—extensor digiti brevis; NR—not recordable; Popl—popliteal fossa.

## Data Availability

The data that supports the findings of this study are available on reasonable request from the corresponding author.

## Abbreviations

CJD: Creutzfeldt–Jakob Disease; PrPSc: isoforms of prion proteins; CNS: central nervous system; PNS: peripheral nervous system; CSF: cerebrospinal fluid; MRI: magnetic resonance imaging; RT-QuIC: real-time quaking-induced conversion; EMG: electromyography; PSA: prostate-specific antigen; WCC: white cell count; VDRL: Venereal Disease Research Laboratory; CMV: cytomegalovirus; HBV: hepatitis B virus; HCV: hepatitis C virus; HIV: human immunodeficiency virus; EBV: Epstein–Barr virus; CT: computed tomography; ENG: electroneurography; IVIG: intravenous immunoglobulins; MMSE: Mini Mental State Examination; DWI: diffusion-weighted imaging; EEG: electroencephalogram; PSD: periodic synchronous discharges; CIDP: chronic inflammatory demyelinating polyneuropathy; SAPs: sensory action potentials; CMAP: compound muscle action potential; TSEs: transmissible spongiform encephalopathies; PrP^C^: cellular prion protein; GSHs: golden Syrian hamsters; CMT: Charcot–Marie–Tooth disease.
